# Polycystic ovary syndrome: new insights on the puzzle of adiposity, chronic low-grade inflammation and metabolic disturbances

**DOI:** 10.20945/2359-3997000000205

**Published:** 2020-03-04

**Authors:** Poli Mara Spritzer

**Affiliations:** 1 Unidade de Endocrinologia Ginecológica Serviço de Endocrinologia Hospital de Clínicas de Porto Alegre Porto Alegre RS Brasil Unidade de Endocrinologia Ginecológica, Serviço de Endocrinologia, Hospital de Clínicas de Porto Alegre (HCPA), Porto Alegre, RS, Brasil; 2 Laboratório de Endocrinologia Molecular Departamento de Fisiologia Universidade Federal do Rio Grande do Sul Porto Alegre RS Brasil Laboratório de Endocrinologia Molecular, Departamento de Fisiologia, Universidade Federal do Rio Grande do Sul (UFRGS), Porto Alegre, RS, Brasil

Polycystic ovary syndrome (PCOS) is a frequent and complex condition affecting women of reproductive age and is characterized by hyperandrogenism, irregular menstrual cycles and polycystic ovarian morphology (PCOM). Women with PCOS also present higher prevalence of obesity, metabolic disturbances and functional perturbations in adipose tissue. Emerging evidence suggests that hyperandrogenism may induce adipocytes proliferation, as observed in women with PCOS. Also, higher percentage of body fat mass and androgen excess have been associated with insulin resistance ( 1 ). Expansion of adipose tissue has been linked to altered secretion of adipokines, inflammation and apoptosis. The hypertrophy of adipocytes and androgen excess in PCOS lead to chronic low-grade inflammation related to compression phenomena in the stromal vessels, local hypoperfusion and disturbed cytokines production. These derangements on the adipose tissue function and secretion may influence the pathophysiology of PCOS through their impact on metabolism and on sex steroid secretion ( 2 ).

In this issue of *AE&M,* Cardoso and cols. ( 3 ) report the associations between adiposity, metabolic traits and adipose tissue-secreted products in a sample of women with PCOS compared to controls. The subjects were stratified according to body fat percentage (BFP) classes and both PCOS and controls were similar regarding age, body mass index (BMI), waist-to-hip ratio, body fat and lipid profile in each BFP class. In turn, considering the same body fat class, insulin-resistance markers (insulin and HOMA-IR) as well as testosterone levels were higher in PCOS compared to controls. Leptin levels were similarly higher and adiponectin levels were lower in both PCOS and control subgroups presenting greater adiposity. In contrast, in these subjects with greater body fat, inflammation markers – TNFα and IL6 levels were higher in PCOS compared to controls. The outcomes of this study, analyzing adipokine levels associated to body fat instead of BMI, expand previous published data about leptin circulating levels in PCOS and controls that indicated a direct relationship between leptin and the amount of body fat, being significantly increased in overweight or obese subgroups, in the presence of PCOS or not ( 4 , 5 ). Conversely, current data are not yet conclusive in relation to adiponectin serum levels, as also cited by Cardoso and cols., and other studies have found lower adiponectin levels in women with PCOS compared with controls ( 6 ). The disagreement among studies may be related to distinct factors, such as ethnicity or to differences in the methods of adiponectin determination. Recently, the measurement of high molecular weight-active adiponectin forms instead of total adiponectin has shown to increase the accuracy of assessments of the relationship between adiponectin and insulin resistance in PCOS ( 7 , 8 ). Interestingly, the results of the Cardoso et al study also reinforce the notion that higher/dysfunctional fat mass in PCOS may release inflammatory mediators, such as TNF and IL6, contributing to pathophysiological mechanisms related to the development of metabolic and reproductive dysfunctions of the syndrome ( 2 , 9 ).

Emerging evidence also suggests vitamin D deficiency or related gene variants may play a role on the endocrine and metabolic disturbances associated to PCOS ( 10 - 13 ). However, even if its nuclear receptor (VDR) is observed in many tissues, including the ovary, the exact mechanism of action of vitamin D has yet to be established in PCOS. Previous trials addressing its circulating levels have shown lower, similar or higher serum concentrations in women with PCOS ( 12 , 14 - 16 ).

In this context, Eftekhar and cols. ( 17 ) sought to examine serum vitamin D levels among the four phenotypes of infertile women with PCOS. A control group consisting of healthy women with a background of male factor for infertility was also included in this retrospective study. The evaluation revealed lower serum vitamin D levels in the PCOS group compared to control women and no differences among the distinct phenotypes of participants with PCOS. It’s noteworthy that BMI was similar among the PCOS sub-groups. This is an interesting approach toward associations between vitamin D and reproductive traits in PCOS and, as the authors suggest, further prospective studies with larger sample sizes and other ethnicities are needed to confirm the present results. In another recent study addressing the prevalence of vitamin D deficiency in women with PCOS and different phenotypes, those presenting androgen excess had higher crude odds ratio for vitamin D deficiency, but the significance was lost after adjustment for BMI and ethnicity ( 18 ). Indeed, considering that obesity has been linked to lower serum vitamin D levels ( 19 ) and women with PCOS present higher prevalence of overweight/obesity as well as insulin resistance, assessing a potential independent role of vitamin D in PCOS still remains a challenge ( 14 ).
